# Maternal Time Use Drives Suboptimal Complementary Feeding Practices in the El Niño-Affected Eastern Ethiopia Community

**DOI:** 10.3390/ijerph19073937

**Published:** 2022-03-25

**Authors:** Asnake Ararsa Irenso, Shiferaw Letta, Addisu S. Chemeda, Abiyot Asfaw, Gudina Egata, Nega Assefa, Karen J. Campbell, Rachel Laws

**Affiliations:** 1 Institute for Physical Activity and Nutrition (IPAN), School of Exercise and Nutrition Science, Deakin University, 221 Burwood Highway, Melbourne, VIC 3125, Australia; karen.campbell@deakin.edu.au (K.J.C.); r.laws@deakin.edu.au (R.L.); 2 School of Public Health, Haramaya University, Harar P.O. Box 235, Ethiopia; 3 School of Nursing, Haramaya University, Harar P.O. Box 235, Ethiopia; shife1973@gmail.com (S.L.); abinahom21@gmail.com (A.A.); negaassefa@yahoo.com (N.A.); 4 Department of Food Process Engineering and Postharvest Technology, Ambo University, Ambo P.O. Box 19, Ethiopia; addisuus@gmail.com; 5 School of Public Health, Addis Ababa University, Addis Ababa P.O. Box 9086, Ethiopia; gudina_egata@yahoo.com

**Keywords:** El Niño, complementary feeding practices, maternal time use, Ethiopia, child malnutrition

## Abstract

Ethiopia is affected by recurrent drought and food-insecurity crises, including El Niño. El Niño started in mid-2014, worsened in 2015, and continued in 2016, leading to a widespread food-insecurity emergency resulting in a surge in the rate of acute malnutrition in infants due to suboptimal feeding practices. This study explored how El Niño influenced complementary feeding practices in the eastern Ethiopia community from March to September 2016. It was an exploratory qualitative study with a basic interpretative qualitative approach. A general inductive approach was used for the analysis. The study involved 11 focus group discussions (FGD) with a total of 76 people, including three with mothers, three with Health Development Army (HDA) leaders, two with fathers, two with traditional birth attendants, and one with religious leaders. El Niño resulted in failed crops and loss of livestock, resulting in reduced dietary diversity and meal frequency. El Niño resulted in suboptimal complementary feeding practices by reducing food access and altering livelihood and coping strategies, reducing the time mothers allocated to child feeding, keeping them away from home, and stressing community health services. The maternal suboptimal time allocation is central to the poor complementary feeding practices. Thus, the women should be supported with climate-resilient livelihood options in their villages, allowing them to feed their children and attend education sessions with HDA leaders.

## 1. Introduction

The complementary feeding period represents a transition from exclusive breastfeeding to family foods at around six months. The promotion of optimal complementary feeding protects children against micronutrient deficiencies, the high rate of growth faltering (irreversible after two years of age), recurrent bouts of infections, and subsequent death [1,2]. Optimal complementary feeding practice is one of the evidence-based early-year interventions for children 6–24 months old, and is of great relevance in developing countries.

In a resource-limited setting, complementary food is prepared at home by using traditional methods. Meeting optimal complementary feeding recommendations by using homemade foods, an extension of family foods in Ethiopia, has remained a challenge [3]. According to the Ethiopia Demographic and Health Survey, a low proportion of children achieve the recommended diversified diet (14%), minimum meal frequency (45%), and minimum acceptable diet (7%) [4]. These practices promote the vicious cycle of low food intake, nutrient deficiency, infection and loss of appetite [5], and the problem is likely to deteriorate with climatic shock.

Maternal and child nutrition improvement is intrinsically linked to sustainable agriculture [6]. However, ongoing climate change continues to influence the food system by reducing crop production, thus altering food availability, food prices, and food supply-chain infrastructure [7]. In developing nations, climatic change since 2014 has either slowed the progress in reducing undernutrition or increased the number of undernourished children [8]. The effect is marked in sub-Saharan African countries, where the rural poor have the least resilience and adaptability to climatic shocks. Subsequently, the climatic change increases inequality, threatening the progress toward sustainable development goals [9].

Ethiopia is affected by a recurrent drought and food-insecurity crises, including El Niño. El Niño is a climatic event marked by the abnormal warming of sea surface temperature in the central and eastern equatorial Pacific Ocean, which causes precipitation anomalies leading to either drought or heavy rain [10]. In Ethiopia, El Niño started in mid-2014 and worsened in 2015, and caused the failure of *kiremt* (the boreal summer that lasts from June–September) rainfall in Ethiopia [11] and the second rainy season of *belg* (which lasts from February–May). During this time, the eastern part of Ethiopia received the lowest level of rainfall in 50 years. As a result, cereal yields were slashed by as much as half, leading to a need for urgent humanitarian assistance for people who lost food, water, and livelihoods [12]. Such climatic shock has been shown to affect the underlying nutrition drivers such as food security, care capacity and health services, water, and sanitation [13].

Even when climatic conditions are predictable, child undernutrition and its consequences are a significant public health problem in eastern Ethiopia. Wasting is unacceptably high [14], increasing the associated high risk of morbidity and mortality [15]. This is likely to worsen for rural children during El Niño and remain above the global goal of five percent [15]. Gillespie and van den Bold (2017) highlighted a disconnect between agriculture and nutrition, in a way that agriculture continued to be both the livelihood and the source of risk for rainfed farmers, the most nutritionally vulnerable rural community. Hence, there is an urgent need for leveraging agriculture for nutrition [16], especially for high-risk rural children [13].

In Ethiopia, child-feeding practices are affected by a range of climatic conditions. In the short term, even good seasonal rainfall leads to suboptimal infant feeding practices by increasing agricultural labor demand, resulting in decreased infant caregiving [17]. On the contrary, in December 2015, El Niño triggered a drought that resulted in 10.2 million Ethiopians requiring emergency assistance. These were in addition to those already receiving support from the Productive Safety Net Programme (PSNP), a program with cash or food transfer for chronically food-insecure households [18]. In such events, there is a surge in acute malnutrition cases among infants and young children, a proxy of the impact of drought on the entire population. Thus, timely, safe, adequate, and appropriate complementary food support is critical to child health and survival [19], and support should consider contexts such as the traditions of child feeding and how the community deals with food insecurity [20]. Given the limited understanding of feeding contexts in Ethiopia during drought, this study aimed to examine how complementary feeding practices are influenced in the food-insecure setting of the eastern Ethiopia community worsened by El Niño.

## 2. Materials and Methods

### 2.1. Study Area and Setting

**Figure 1:**
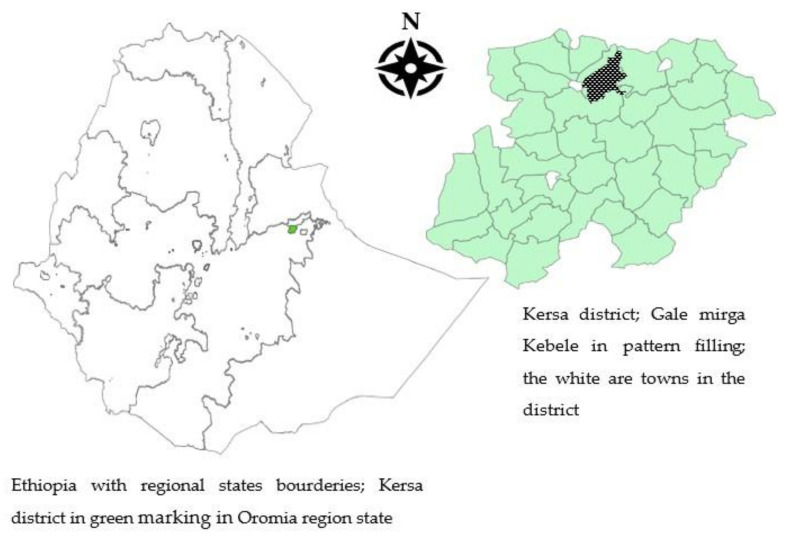
The map of the study settings of Gale Mirga kebele, Kersa district, eastern Ethiopia.

The current study was conducted in Gale Mirga *kebele* (the lowest administrative unit in Ethiopia) of Kersa district of eastern Ethiopia from March to September 2016 (Figure 1). Kersa district has 35 kebeles; each comprises approximately 1000 households. The setting was selected as it was one of the food-insecure districts of the eastern Hareghe zone and the hotspots of El Niño. According to the 2014 nutrition causal analysis, the district’s general acute malnutrition and severe acute malnutrition rate was 7.5% and 0.8%, respectively. The availability of complementary foods was also suboptimal, with 73% of the main food prepared coming from sorghum and 19.9% from maize, reflecting food insecurity and the utilization of available resources at home [21]. According to Kersa Demographic and Surveillance data, the district crude birth and death rates are 37.2 and 7.8 per 1000 population, respectively. The district infant and under-five mortality rates were 46.9 and 77.4 per 1000 live births, respectively (Aseffa et al., 2015). The mortality figures are comparable to national-level infant mortality (48 per 1000 live births) but higher than national under-five mortality (67 per 1000 live births) rates [4]. The Sustainable Development Goal has set a target to reduce the under-5 mortality rate to less than 25 per 1000 live births [22].

### 2.2. Study Design

This study was an exploratory qualitative study with a basic interpretative qualitative approach [23] to uncover how the El Niño influenced complementary feeding practices.

### 2.3. Study Participants and Inclusion Criteria

Participants were selected purposefully to represent diverse community roles and first-hand experiences of the effects of El Niño. To be included in the study, participants had to have one or more of the following roles:

- Health Development Army leader (a volunteer woman who leads a group of 25 to 30 women responsible for delivering health messages in rural Ethiopia) for their insights from a education provider perspective;
- Mother or father of children less than two years of age for their insights into the direct impacts of El Niño on caregiving and child-feeding practices;
- Traditional birth attendant who has insights into cultural knowledge related to child feeding; and
- Religious leader for their unique insights into community problems.

#### Participant Recruitment

All HDA leaders, traditional birth attendants, and religious leaders in the district were invited to participate in the study. Mothers and fathers of children were invited based on their proximity to the meeting place. In recruiting participants, a letter of support was sent to the Kersa district health office that linked the research team to the health-extension workers of the Gale Mirga kebele. The health-extension workers provided the research team with information on the eligible study participants, which informed the sampling procedure and venue for focus group discussions (FGDs). Separate FGDs were conducted for each group. Data collection and analysis took place without deciding a priori the sample size, i.e., data collection went on until the ongoing inquiry revealed no new data.

### 2.4. Data Collection

Focus group discussions were held at local primary school classrooms, which provided a quiet and private environment. Each FGD was facilitated by two researchers of Haramaya University who had Master’s Degree qualifications, previous experience of moderating FGD, and excellent native language (Afaan Oromo) proficiency. Focus groups were audio-recorded and observed, and a moderator took notes. The participants informed the plain language summary of the study objective and procedures, benefits, and risks of participation and their rights and obtained consent ahead of the data collection.

At the start of a focus group, a moderator acknowledged the presence of the audio-recording equipment, assured participants of confidentiality, and allowed people to withdraw if they were uncomfortable with being audio-recorded. Concerning the researchers’ position in this study, Haramaya University has a Demographic and Health Survey setting in the district, but not in this specific kebele; hence, investigators had less knowledge about the study setting. Researchers were vigilant to a predisposition to their previous knowledge of the study settings on the study, if any.

The sociodemographic data of participants were collected. Participants were asked about locally produced foods, those included in complementary foods and how complementary feeding practices are affected by household food insecurity related to El Niño. Although the communities were affected by chronic food insecurity from the failed rainy season, a distinction was made with El Niño using the information on severity and duration of most extreme events predicted by the Famine Early Warning System Network [24]. Moreover, strategies used to manage household food insecurity and its relation to complementary feeding practices were inquired, taking into account variations across the FGD participants.

### 2.5. Data Analysis

The FGD’s audio recordings were transcribed verbatim and translated into English by the research team, who were fluent in the native language and in English [25]. The authenticity of the transcripts was verified by two authors who moderated the discussion. The two authors read and discussed the transcripts and compared them against the original recording. The data collection and analysis were conducted simultaneously to identify areas for further exploration in subsequent focus groups. A general inductive approach was used for the analysis [23]. The final version of each transcript was imported into ATLAS. ti 7 Windows for coding by the lead author and another researcher [26]. The initial codes were reduced into sub-themes and themes by bringing together the experience of the FGD participants.

## 3. Results

A total of 76 people participated in the study, across eleven groups, including three FGDs with mothers, three FGDs with Health Development Army leaders, two FGDs with fathers, two FGDs with traditional birth attendants, and one FGD with a religious leader. Almost all study participants were farmers, and petty trading was a frequent alternative income-generating activity for HDA leaders, birth attendants, and mothers. One in five participants (a quarter of participants) were fathers and mothers of children less than two years of age, respectively (Table 1).

The typical complementary foods of the community were traditional family foods (with/without modifications). The typical child food-modification measures mentioned by participants included mixing local ingredients such as maize or sorghum, mashing, and adding extra oil, butter, fruits, or vegetables. However, mothers did not implement these modifications routinely. The family food modification and quality enhancement depended on the season and the child’s health. While seasonality influences the availability and access to foods, which had been severely affected by El Niño, child illnesses and associated health-facility visits had enabled counseling on feeding practices and family food modification. A 23-year-old mother with an 18-month-old child said:

*“A different food is prepared when a child gets sick or malnourished. I buy potatoes, carrots, beetroots, cabbages, and other vegetables when only the child gets sick. Even during illness, the varieties of the foods I give the child depends on the seasonal availability.”*

For complementary foods, caregivers typically limit ingredients to one food group, including grains, roots, and tubers, rather than diversifying by adding other plant and animal-source foods. The mothers attribute the current composition of their child’s diet to recurrent and prolonged drought and associated water insecurity from El Niño. A 32-year-old mother of a 7-month-old child said:

*“The child feed on family foods after six months. I give him injera, a cereal-based food made from sorghum with some stew. We have been given orientation on how to prepare child foods from 16 ingredients of all varieties. However, we cannot afford it. Therefore, we make child food with limited ingredients. The better-off family’s babies can have bottle-feeding.”*

**Figure 2:**
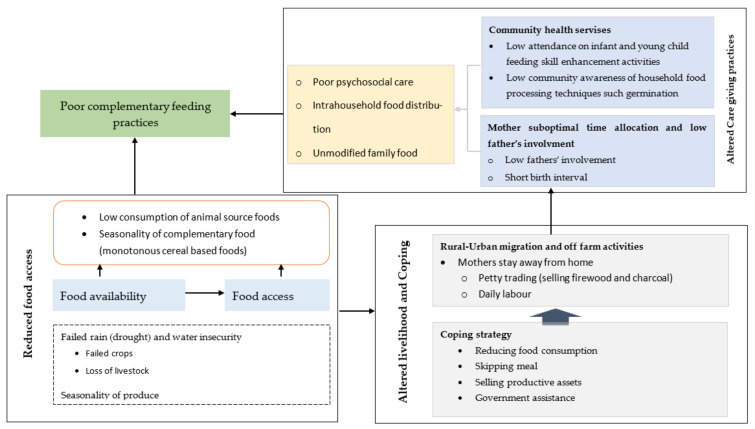
Factors that affect complementary feeding practices in El Niño affected community of *Gale Mirga kebele* of Kersa district 2016.

With this background, the analysis resulted in three themes, and their relationship with child suboptimal complementary feeding is shown in Figure 2. These themes included reduced food access, altered livelihoods and coping strategies, maternal time use and fathers’ involvement, and community health service availability.

### 3.1. Suboptimal Complementary Feeding from Limited Food Access

All study participants indicated that the district, specifically their kebele, had been affected by a variable degree of seasonal food insecurity and mentioned that the selected households had benefited from the Productive Safety Net Program (PSNP). The PSNP provides predictable food and cash transfer and livelihood protection to the most disadvantaged households, and in return, the household members contribute labor to the environmental protection activities. However, the level of food insecurity associated with El Niño was worse due to the previous years’ droughts. The cumulative water insecurity due to El Niño had resulted in the failed crop and livestock loss, which directly reduced child food intake.

The participants agreed that including animal-source foods for a child is a challenge, which was affected by the livestock loss due to recurring drought and El Niño. The community key informants raised the issue of how the introduction of improved agricultural products to their community gave them the hope of regaining their villages’ productive assets. A 62-year-old religious leader said:

*“Our villagers used to have cows, milk and milk products, sheep, and small-scale poultry. Though some households still own some, the last four-drought years have brought deadly livestock diseases that resulted in a significant loss, hence access to animal-based foods. Haramaya University has provided us with four heifers and a bull for our village.”*

The participants also viewed the variability of food available throughout the year and large family size as primary reasons for the persistent pattern of undernutrition in their village. A 45-year-old HDA leader said:

*“The main harvest season of our major food crop, sorghum, is in February. We rely on it for three to six months (June). However, the last three years were tough. We purchase food for most months of the year. Previously, lower levels of child undernutrition occur which peak from July to September. Now, large numbers of children are affected by undernutrition throughout the year.”*

### 3.2. Altered Livelihood and Coping Strategy

The other significant discussion was focused on how food insecurity associated with El Niño has gradually normalized the coping strategies used, such as reducing food consumption, reducing meal frequency, selling productive assets, and seeking urgent government assistance. When asked how children are affected by these strategies, participants indicated that children also share the coping behavior of reduced food consumption and frequency because of availability/food-access issues and because parents stay away from home. A religious leader described his own experience as follows:

*“The Khat, the primary cash crop in our community, has dried from prolonged drought. Besides, food produced from my ever-decreasing plot of land is estimated at two quintals, and it cannot sustain my nine household members. So, like any other communities members, I travel to the nearby cities/towns to work as a daily laborer. Some people get government assistance, and in the current state, most people need it.”*

Another religious leader said:

*“We eat twice a day, in the morning and evening. The children also eat less and act fussy due to a lack of regular feeding. When children become irritable, they are given little food to calm them down.”*

An HDA leader said:

*“In difficult seasons, we sell our cows, even when they are about to have a calf, sell our sheep and goats to buy food. Families who do not have these assets work as daily laborers by travelling to nearby towns like Dire Dawa, Awaday and Harar.”*

The mothers frequently went to the nearest town/cities (Dire Dawa city and Kersa town) for petty trading, including selling firewood and charcoal produced locally and returned home in the evening. On the other hand, men (older boys and fathers) work as daily laborers, stay and work in urban areas, and only visit the family periodically. Participants also reported that both sexes share petty trading and daily labor activities. In the absence of the parents, the child care responsibilities are delegated to extended family members and girls. Temporary rural–urban migration was identified as a barrier to maternal and child care giving, as described in the following. An HDA said:

*“Children have a recurrent illness with swelling of their body. The health professional told us a food shortage caused it and gave us plumpy nut (a supplementary therapeutic food for malnourished children). The child gets undernourished because, in our community, the women leave the child at home in the morning and return in the afternoon to bring some food for the family.”*

Another HDA said

*“Family members staying away from home (males) come home to nearby towns visits their family frequently when they are daily laborers or monthly when they are employed on a monthly basis.”*

Another 35-year-old father said:

*“If the mother is away from home, my first-born child girl is in charge of child care. She prepares and feeds the child. In my previous experience, my mother was living with me, and she used to care for my older child. However, now my daughter can handle it. I have never tried preparing the child food.”*

### 3.3. Maternal Time Use and Father’s Involvement

The coping and livelihood strategies related to El Niño has altered women’s time use. The maternal time allocation for child feeding was negatively influenced by staying away from home for work and the short birth intervals that resulted in mothers needing to care for multiple young children. Furthermore, fathers had low involvement in child care due to temporary absence from home, e.g., because they are going to cities for work, and even when they are home, they do not typically look after children. The limited role of fathers in caring for children has put the childcaring burden disproportionately on the women; hence, it costs the mother more time. A mother of 7 month-old children said:

*“Compared to males (household heads), who have a better opportunity to eat first at home or get food from somewhere else, women usually serve their family breakfast at 7 AM, leave the house to bring food, return at 1 PM, breastfed their child, and prepare the food. Other family members could lend a hand to the women.”*

A 47-year-old HDA said:

*“Children like to get food regularly, at 10 AM, 11 AM, 12 AM, 3 PM, and more. Mothers do not afford to feed this way. We used to feed children milk and eggs in good years, which they could no more do. Mothers neither prepare separate food for children as they leave home in the morning and return in the afternoon. Time limit them.”*

The livelihood strategy to mitigate the influence of El Niño has led to a low maternal time allocation around child care and negatively influenced participation in community-based complementary feeding activities. The mothers are often away working/trading when health-extension workers and the Health Development Army conduct nutrition educational activities. When the mother is available, the focus of nutritional education had limited complementary food-preparation demonstrations.

Concerning the males/fathers’ involvement during the El Niño-triggered drought, they neither attended nor actively supported the process of infant and young-child feeding activities, including the community-based food preparation demonstrations as they stay away from home to support their family. The low level of direct male involvement influences the initiation of complementary foods, including the early provision of butter and sweets such as biscuits and the over-reliance on unmodified family foods at an early age. A 40-year-old father said:

*“Child feeding is the primary responsibility of a woman. The male has to work and bring food home. But, he does not have anything to bring from the farm. I believe my child should be fed when hungry; hence, I probably ask the mother whether the child has had food or tells the mother to feed the child if the child cries. Otherwise, I have never cooked the child food nor attended its preparation at home or publicly.”*

A 60-year-old traditional birth attendant said:

*“During and after pregnancy, the husbands do not bother whether the women/ the child have food. Unless a woman cares for herself, she does not get suggestions on the food. However, husbands are supposed to ask about the food and should have cared more.”*

Another father, a 32-year-old, also said:

*“I have two kids. When my wife gives birth, I support her, and she gives what the child needs. The child gets only if the women get. For instance, if the woman drinks water, the child gets water; if the woman gets balanced food, so is the child. One thing that we can’t erase from our minds is giving water early, shortly after birth and butter from around four months.”*

Participants agree that mothers disproportionately shouldered the effect of El Niño-triggered food insecurity, the coping strategy, along with their reproductive role and child care. Women’s many roles collectively led to suboptimal time allocation between child feeding and household chores and on-farm and off-farm activities. Participants agreed that women are not available at their homes in the afternoon to bring food for the family. The mothers acknowledge that their absence and low level of fathers involvement affects the overall child feeding practices and intrahousehold food distribution. Older children are eating more of the food meant for younger children. A 29-year-old mother of a 12 month-old child said:

*“In the afternoon, we go to the market or farm to protect crops from wild animals such as monkeys; hence, we do not have time to prepare child foods. Instead, older children look after the child, prepare and feed in the afternoon, and we prepare the food in the evening. One of the concerns for our absence is that older children share child food.”*

A traditional birth attendant said:

*“Every household has its own problems.… Women have extra burdens. Family members expect us to do everything. Even husbands do the same.”*

### 3.4. Community Health Services Circumstances during El Niño

Discussion surrounding the community health workers involvement has stimulated experiences related to nutrition activities during pregnancy and delivery during El Niño-triggered drought. The community recognizes the positive role of community health workers in birth preparedness and institutional delivery. The Health Development Army support and nudge women to give birth at health facilities, giving an excellent opportunity to counsel the mother on infant and young-child feeding, increasing birth spacing, and other maternal and child health care services. Still, participants mention the short birth interval as a primary concern contributing to child undernutrition in the community. A 55-year-old who was previously a traditional birth attendant, and is now a Health Development Army leader said:

*“All women must give birth at the health facility. We encourage and support them to start breastfeeding immediately. …Concerning colostrum, there are two types of colostrum. The one that older babies get from their pregnant mother [with a narrow birth spacing] and the newborn gets from his mother at birth. The one that the baby feeds on after birth is clean and good. The one that the child feed on while the woman is pregnant has water that causes swelling of the head and nutritional deficiency.”*

Parents and critical informants agree that HDA leaders are a group that governmental and non-governmental stakeholders primarily select to get capacity-building activities, including training and creating awareness about infant and young-child feeding. HDA leaders cascade acquired skills back to their community members. However, cascading of acquired knowledge and skills and applications of improved practices are limited or short-lasting from limited access to necessary food groups and from drought. A 42-year-old HDA leader said:

*“I have received orientation in multi-mix complementary food preparation and shared it with women in my group. However, they use it briefly and stop it due to lack of awareness, necessary ingredients, lack of knowledge and skills, and other women are tired of processing it separately from family foods.”*

The community has felt the high burden of undernutrition, and its consequences during El Niño-triggered drought. The problem has created a massive demand for interventions, and the community members think that the problems are beyond the capacity of the local health workforce, such as health-extension workers. A 39-year-old Health Development Army leader and a mother said:

*“The children get malnourished both when we feed breastmilk alone and also after we start giving other foods. If we have two young children in the same house and feed them the same type and amount of foods, one can get malnourished while the other remains normal. The malnourished child gets better after receiving the treatment. So, I think the problem might be with the mother. If the family failed to provide the child with better foods, the child would return to the clinic with malnutrition within three or four months.”*

## 4. Discussion

This study aimed to qualitatively explore how complementary feeding practices are influenced in the food-insecure setting of the eastern Ethiopia community impacted by El Niño. Central to young children’s suboptimal complementary feeding practices was the mothers’ reduced time allocation for child care and feeding. Mothers working away from home and short birth spacing reduced the time mothers spent with their young child. Maternal absence from home was also a barrier to participation in community-based nutrition and health educational activities. There was a lowlevel of fatherly involvement in the child care.

Optimal time allocation is a critical component of caregiving. According to the UNICEF conceptual framework, care is defined as “the provision in the household and the community of the time, attention, and support to meet the physical, mental, and social needs of the growing child and other household members” [27]. In this regard, while maternal time allocation has an indirect path to child undernutrition, suboptimal complementary feeding directly leads to undernutrition [28]. The World Health Organization recognizes, recommends, and advocates for governments to have supportive regulation for formal institution working mothers to get maternity leave, facilities, and time for breastfeeding in the workplace [29].

Accordingly, the revised Ethiopia labor law grants female employees one month of paid pre-natal and three months of post-natal leave. That is a minimum of 120 days of maternity leave, a probation period of 60 days, and no paternity leave [30]. However, rural Ethiopian women, who are engaged in work outside of formal institutions but are paying taxes and contributing to the economy through informal sectors, neither benefit from maternity leave nor have similar schemes in place. The absence of a systematic strategy to keep the rural child with its mother must be acknowledged and addressed in an environment where traditional motherly roles change continually. Women’s roles have changed with El Niño, making them financially vulnerable and absent from the child-caring role, and in considering child health and malnutrition, the government must address this change in roles [4]. Otherwise, such a situation will continue to represent a source of inequity in Ethiopia [31].

Maternal time allocation is an essential modifiable child-feeding factor that can improve feeding behaviors [32,33], and enhanced social support is an integral part of it [34,35]. The UNICEF conceptual framework of child care considers the time a household and the community has dedicated to the child’s well-being (UNICEF, 1991). The maternal time allocation determines whether the mother is with the child or away during the day. In both cases, while preparing and feeding complementary foods is the mother’s primary responsibility, the maternal child care time allocation is shared among household members such as fathers, grandparents, siblings, and non-relatives [31]. Based on the shared role, time allocation to child care varies depending on the following factors: whether two parents or a single parent is present, whether one child or two children seek close attention, and whether non-parents are delegated to look after the child or children [31,36].

In this regard, our study showed the delegation of young-child feeding to non-maternal caregivers’, especially older siblings in the household when the mother is away from home. Similarly, a study conducted in Congo recognizes the mother as the single main contributor for child feeding, but less than the combined care provided by fathers, grandmothers, aunts, siblings, and cousins [37]. Although the Ethiopian infant and young-children feeding initiative recognizes the importance of supporting partners and family members [38], there is limited evidence of its implementation. Given the critical roles non-maternal caregivers play in child care, it is vital that policy address this as part of a comprehensive nutrition plan.

Our study showed minimal fatherly involvement in child feeding with or without prolonged work and time spent away from home during drought, and the cultural norm with regard to a father’s role is mainly limited to income generation [39]. The maternal time allocation and competing demands determine whether the mother is with the child or away during the day, and who feeds the child complementary foods [31]. The male involvement in household work and child-rearing empower the women by alleviating competing demands on her time [40]. Ethiopia considers gender mainstreaming as a cross-cutting nutrition subject, and male involvement in community-level nutrition is part of it. The low level of fatherly involvement in child feeding implies problems of translating the nutrition policy that considers the role of both mothers and fathers in child feeding; in addition, there is a continued, uneven burden that mothers have in the child care and feeding [38].

Contrary to our findings, evidence from southwestern Ethiopia shows high paternal involvement with their presence at home, finance, child care, and feeding [41]. The difference might be related to stable local livelihood during lean seasons that keep the fathers close to the family, and the expectations of fathers might be different from our study population. The paternal economic stability reduces the risk of a severe form of a coping strategy such as reducing meal frequency and amounts. This gives the mother the freedom of child-feeding decisions, food procurement, and preparation [42]. The study conducted in southwestern and northern Ethiopia also showed the favorable implications of fatherly involvement in child feeding [41,43] and better linear growth after controlling for food-security status [41].

Giving sufficient time for child care is essential but not sufficient to make a difference in the quality of feeding practices. For instance, Jain and Zeller (2015) reported that looking after children full-time makes no difference in children’s food consumption compared to the maternal time spent outside the home, which has been shown to have a positive effect on boys’ food consumption [44]. This implies that there is a need to balance maternal time allocation with livelihood activities. The balance can be achieved by empowering the women economically through work opportunities close to home and enabling them to use available resources through nutrition skills [38,45].

Female economic empowerment can be in the form of alternative climate-resilient livelihoods that keeps the mother with her child in her village. Such economic opportunities drive an equitable share of child caregiving between men and women, resulting in fewer time constraints. Furthermore, economic empowerment helps women hold and control property and contribute to the child care expenditure similar to men [46]. According to the United Nations Development Programme, economic empowerment should be tailored to the women’s needs, settings, such as rural areas, and vulnerabilities, including recurrent climatic shocks and food insecurity [47]. In line with this argument, evidence from 12 African countries showed a positive association between the economic dimension of women’s empowerment and meeting the recommendations for infant and young-child feeding [48].

In our study, participants elucidate the negative influence of narrow birth spacing on child nutrition similar to the Ghanian connotation of the *kwashiorkor*, a nutritional problem a weaned child gets when a younger sibling is born [49,50]. The fact that the problem is usually observed after six months of age underscores the interaction of food scarcity, limited access to clean water, and calamities such as drought [50] with complementary foods. Indeed, a recent systematic review and meta-analysis in Ethiopia show the pooled prevalence of short birth spacing, an interval of less than 24 months at 46.9%, which is associated with shorter breastfeeding duration [51]. Addressing this problem is important to tackling malnutrition and achieving sustainable Development Goal 2, zero hunger [52], by enhancing family planning services via focusing on reducing fertility and increasing birth spacing [53,54].

Our study had several strengths, such as telling a story from people directly affected by El Niño and illustrating previously unreported beliefs of child-feeding practices in our study context. The findings of this study, while not generalizable beyond the study setting, may help explain the negative implications of adverse climatic conditions on complementary feeding practices of similar food-insecure communities. This study has some limitations. First, our conclusions and interpretations relied on the participants chosen for the study; hence, we might be missing information from other stakeholders, such as district and kebele officials and healthcare workers.

## 5. Conclusions

The maternal time allocation is central to suboptimal complementary feeding practices in El Niño-exacerbated food-insecure settings of eastern Ethiopia. A key implication of our findings is that women should be supported with climate-resilient livelihoods options in their villages, allowing them to be close by to feed their children and attend relevant education sessions. Furthermore, the birth-spacing issue can be addressed by improving family planning services, particularly by increasing the minimum birth interval to more than two years. Low paternal involvement requires understanding and addressing perceived norms. Future research can inform the policy on the “how” aspect of addressing males’ behavior in the broader social and cultural context, messages that work, and platforms to deliver the message to expand fathers’ roles to include child-feeding responsibilities.

The focus of HDA and health-extension workers should be repositioned from conveying oral nutrition messages to skill-based activities so that mothers are equipped with an adaptive capacity to handle El Niño like events by using existing resources. At this juncture, it would be useful to understand why nutrition-related practical activities are featured so little in current health-promotion offerings, and the opportunities and challenges for delivering these skills through the HDA structure.

## Figures and Tables

**Table 1:** Background information of research participants.

Variable		Frequency
Sex	Male	25
	Female	51
Age	18–25	20
	25–35	34
	More than 35	22
Study Participants	Women development army leaders	23
	Mother of children less than two years	20
	Fathers of children less than two years	15
	Traditional birth attendants	11
	Religious leaders	7

## Data Availability

The data that support the findings of this study are available with the principal investigator upon request.
